# Corrosion Resistance of Nanopowders of Borides and Carbides of IV–VIB Group Metals in the Nickeling Electrolytes

**DOI:** 10.1186/s11671-017-2223-5

**Published:** 2017-07-12

**Authors:** Dmytro Shakhnin, Viktor Malyshev, Nina Kuschevskaya, Angelina Gab

**Affiliations:** University “Ukraine”, 23 Lvivska St., Kyiv, 03115 Ukraine

**Keywords:** Corrosion resistance, Nanopowders, Borides, Carbides, Nickeling electrolytes, IV–VIB group metals

## Abstract

The corrosion resistance of nanopowders of borides and carbides of metals of IV–VIB groups, as well as of silicon carbide, was studied in the standard nickeling electrolytes. As objects of study, nanopowders with the content of the main phase 91.8–97.6% and with the average particle size 32–78 nm were used. Their corrosion resistance was evaluated depending on the acidity of the electrolyte, temperature, and duration of the interaction. It was found that, by the corrosion resistance in the electrolytes solutions, nanopowders of borides and carbides within each group of compounds are similar and characterized by unlimited period of induction in alkaline media. An exception is the nanopowder of silicon carbide which is resistant to the solution of any acidity.

## Background

The corrosion resistance of powder materials used as reinforcing phases in composite electrochemical coatings (CEP) is an important characteristic defining the fundamental possibility of their obtaining. Dissolution of powders in electrolyte solutions leads to deterioration of the electrolysis conditions which imposes significant process limitation on the use of each specific material for the CEP obtaining [4, 5, 7]. Analysis of available data shows [8] that a number of studies in which no dissolution of hardening phases (borides) were taken into account contain inaccuracies, and the neglecting of this fact by the author of [6] led to excessively wide advertising of dispersion hardening processes realized in chroming electrolytes containing zirconium diboride. Therefore, study of corrosion resistance of powders of refractory compounds is an important task, and investigation of their nanostates poses a scientific problem as well. Urgent need for such research is also due to the lack of information on this subject. Only in [2], there is evidence of stability in acid solutions of nanostructured nitride-boride composites of titanium and zirconium.

This paper is devoted to the investigation of the corrosion resistance of nanopowders of borides and carbides of zirconium, titanium, vanadium, chromium, molybdenum, and tungsten in the nickeling electrolytes depending on the acidity of the electrolyte, temperature, and duration of the interaction.

## Methods

Test objects were nanopowders of borides and carbides of zirconium, titanium, vanadium, chromium, molybdenum, and tungsten, and also silicon carbide, manufactured by plasmochemical and by high-temperature electrochemical synthesis methods. Main characteristics of the test objects are shown in Table 1. Study of resistance of nanopowders of refractory metals borides and carbides was performed in standard nickeling electrolytes (Table 2).

**Table 1:** Main characteristics of nanopowders of borides and carbides

Compound	Contents^a^ of main phase, %	Avg. particle size, nm	30–70 nm fraction content, %
ZrB_2_	91.6	41	85.1
TiB_2_	92.1	39	77.3
VB_2_	93.3	38	79.0
CrB_2_	96.8	41	82.0
MoB_4_	91.8	62	81.6
WB_4_	97.6	68	82.3
ZrC_0.90_N_0.06_	94.4	41	78.0
TiC_0.90_N_0.06_	91.7	58	81.0
VC_0.85_N_0.05_	94.8	45	76.0
Cr_3_(C_0.80_N_0.20_)_2_	95.6	42	80.0
Mo_2_C	97.2	78	79.6
WC	97.1	76	82.4
SiC_0.95_N_0.05_	96.3	62	75.0

^a^After enrichment

**Table 2:** Compositions of electrolytes, kg/m^3^

Electrolyte	NiSO_4_·7H_2_O	H_3_BO_3_	NaCl	NaF	NiCl_2_·6H_2_O	pH
1	245	30	20	6	–	4.0–5.5
2	300	30	–	–	60	2.0–4.0

The acidity of the electrolyte was adjusted by adding concentrated sulfuric acid. Concentration of powders of carbides and borides was in all experiments 10 kg/m^3^. Prior to treatment in the electrolyte, powders were subjected to repeated refinement, thus reducing the content of nanoscale graphite and boron down to 0.1–0.3% (by weight), and to vacuum thermal stresses to prevent coagulation of the particles. Nanopowder corrosion resistance was evaluated depending on the acidity of the electrolyte, temperature, and duration of the interaction. Dissolution rate was calculated by the insoluble residue mass and by the concentration of ions of carbide(boride)-forming element in the electrolyte determined by magnetometric method [3].

## Results and Discussion

**Fig. 1:**
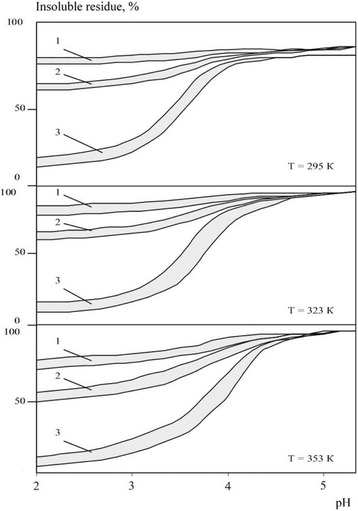
Insoluble residues ratios areas for nanopowders of borides of zirconium, titanium, vanadium, chromium, molybdenum, and tungsten in electrolytes solutions of different acidity depending on the temperature and exposure time *τ* = 1–3 h, 2–24 h, 3–240 h

**Fig. 2:**
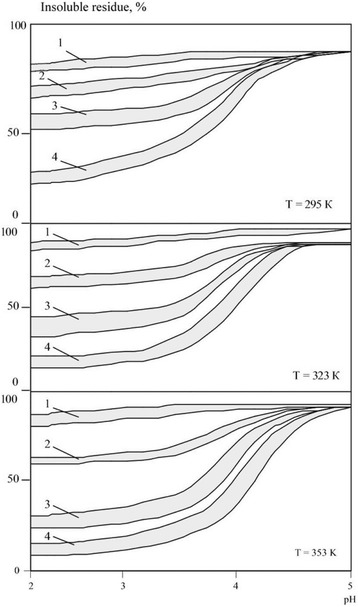
Insoluble residues ratios areas for nanopowders of carbides of silicon, zirconium, titanium, vanadium, chromium, molybdenum, and tungsten in electrolytes solutions of different acidity depending on exposure time and temperature *τ* = 1–3 h, 2–24 h, 3–120 h, 4–360 h

The corrosion studies’ results for nanopowders of borides and carbides are shown in Figs. 1 and 2. It was noted that, in both groups of compounds, corrosion resistance of materials was comparable and attributable primarily to the electrolyte acidity. Therefore, all the obtained corrosion resistance data are better to be presented graphically as ranges into which all the studied materials’ sample curves are fit. In acid electrolytes (pH = 2.0÷3.0), all materials nanopowders were quickly dissolved. For example, after 3 h at *T* = 323 K, boride dissolution degree was 15.6–9.5%; after 24 h, 38.2–31.0%; and after 240 h, 89.9–75.1%. Nanopowders of metal-like carbides have slightly higher corrosion resistance; their dissolution degrees similar to the respective borides were achieved after 24, 120, and 360 h, respectively. All the materials exhibit corrosion resistance drop with temperature increase. It should be caused by the increase of rates of reactions between studied nanomaterials and electrolytes’ acids with the temperature increase.

For all the nanomaterials under investigation, the increase of the specific surface area during the dissolution is also a characteristic. With the same particle shape, their experimentally found specific surface areas rose from 2000 m^2^/kg before treatment up to 10,000 m^2^/kg after it, showing mainly layered nature of the dissolution process. The only exception is silicon carbide nanopowder which degree of dissolution in the whole studied pH and temperature range did not exceed 7–10%.

**Fig. 3:**
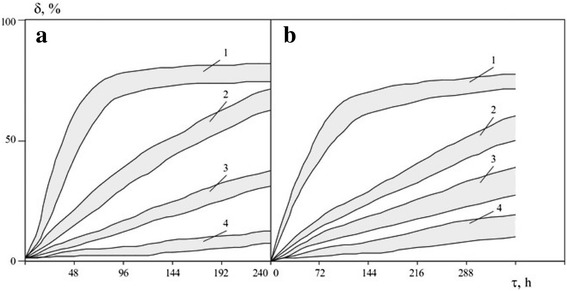
Dissolution degree values areas for nanopowders of borides (**a**) and carbides (**b**) of zirconium, titanium, vanadium, chromium, molybdenum, and tungsten in electrolytes solutions: *T* = 323 K; electrolyte pH value—2.5 (*1*), 3.0 (*2*), 3.5 (*3*), and 5.0 (*4*)

Kinetic curves of dissolution of borides and carbides calculated from change of concentrations of ions of boride(carbide)-forming metals are shown in Fig. 3. Induction periods calculated from the obtained results (i.e., time in which half of the original particulate material is dissolved), with pH 2.5 electrolytes, were within 32÷49 h for borides and within 68÷88 h for carbides; with pH = 3.0 electrolytes, 92÷112 h and 138÷167 h, respectively; and with pH = 5.0 electrolytes, they were practically unlimited. Comparison of kinetic parameters with known data for the coarse powders shows that the dissolution rate of nanopowders is 3–5 times higher.

Thus, corrosion resistance of borides and carbides of zirconium, titanium, vanadium, chromium, molybdenum, and tungsten in the electrolytic solutions within each group of compounds is similar and mainly determined by the acidity of the medium, wherein the nanopowders dissolution rate significantly higher than that for coarse-grained materials [1], which can be considered as one of the manifestations of the size effect. To a lesser extent, the latter is manifested during dissolution of silicon carbide nanopowder resistant within almost all the investigated pH range. Consequently, nanopowders of borides and metal-like carbides may be used in processes of the composite reinforcement with weakly acid or alkaline electrolytes, and of silicon carbide, in processes involving electrolytes of any acidity.

## Conclusions

1. It was shown that the corrosion resistance in standard nickeling electrolytes for nanopowders of silicon carbide, as well as for zirconium, titanium, vanadium, chromium, molybdenum, and tungsten borides and carbides, depends on the acidity of the electrolyte, the temperature, and the treatment duration.
2. It was found that the corrosion resistance values for studied compounds are determined by the acidity of the electrolyte. Rather, quick dissolution of nanopowders in acid electrolytes (pH = 2.0…3.0), reaching 75…90% after 240 h and accelerating with increasing temperature, was noted.
3. Silicon carbide nanopowder is characterized by high corrosion resistance; its dissolution degree does not exceed 8–12% within the entire studied ranges of pH (2.0– 5.0) and temperatures (295–353 K).

## Abbreviation

CEP: Composite electrochemical coatings

## References

[1] Kosolapova TY (1986). *Svoystva, poluchenie i primenenie tugoplavkih soedineniy: Spravochnik* (Properties, preparation, and use of refractory compounds: Reference).

[2] Krastiņš LA, Tsielen UA, Bondars BYa (1982) *Elektroliticheskie kompozitsionnyie pokryitiya na osnove nitrozo bornyih kompozitsiy titana i tsirkoniya* (Electrolytic composite coatings based on boron nitroso compositions of titanium and zirconium). In: Sb. nauch. tr. IPM AN USSR, IPM AN USSR, Kiev, p. 16–21

[3] Krutskij JuL, Djukova KD, Antonova EV, Bannov AG, Sokolov VV, Pichugin AJu, Maksimovskij EA, Uhina AV, Krutskaja TM, Neckina OV, Kuznecova VV (2015) *O korrozionnoj stojkosti vysokodispersnyh poroshkov karbidov nekotoryh perehodnyh metallov* (Corrosion resistance of carbide fine dispersed powders of some transition metals) Science bulletin of Novosibirsk State Technical University 58:271–281

[4] Kuzmar I, Lanin V (2006). *Kompozitsionnyie galvanicheskie pokryitiya* (Composite galvanic coatings). Tehnologii v elektronnoy promyishlennosti.

[5] Ploof L (2008). Electroless nickel composite coatings. Advanced Materials and Processes.

[6] Podchernyaeva IA, Astakhov EA, Umanskii AP, Panasyuk AD, Konoval VP, Panashenko VM (2010) *Struktura i fazovyj sostav detonacionnyh kompozicionnyh pokrytij na osnove TiCrB*_*2*_*i ZrB*_*2*_ (Structure and phase composition of detonation composite coatings based on TiCrB_2_ and ZrB_2_) Powder Metallurgy and Metal Ceramics 49:295–303

[7] Saifullin RS (1977). *Kompozitsionnyie pokryitiya i materialyi* (Composite coatings and materials).

[8] Saifullin RS (1983). *Neorganicheskie kompozitsionnyie materialyi* (Inorganic composite materials).

